# Translational Science and Evidence-Based Healthcare: A Clarification and Reconceptualization of How Knowledge Is Generated and Used in Healthcare

**DOI:** 10.1155/2012/792519

**Published:** 2012-02-14

**Authors:** Alan Pearson, Zoe Jordan, Zachary Munn

**Affiliations:** The Joanna Briggs Institute, The University of Adelaide, North Terrace, Adelaide, SA 5005, Australia

## Abstract

The importance of basing health policy and health care practices on the best available international evidence (“evidence-based health care”) and on translating knowledge or evidence into action (“translation science” or “translational research”) is increasingly being emphasized across all health sectors inmost countries. Evidence-based healthcare is a process that identifies policy or clinical questions and addresses these questions by generating knowledge and evidence to effectively and appropriately deliver healthcare in ways that are effective, feasible, and meaningful to specific populations, cultures, and settings. This evidence is then appraised, synthesized, and transferred to service delivery settings and health professionals who then utilize it and evaluate its impact on health outcomes, health systems, and professional practice. Many of the common theories that address this translational process place it apart from the evidence-based practice cycle and most recognise only two translational gaps. This paper seeks to clarify the nature of evidence-based healthcare and translation science and proposes a reconceptualization that both brings together these two dominant ideas in modern healthcare and asserts the existence of a third fundamental gap that is rarely addressed the gap between knowledge need and discovery.

## 1. Introduction

The challenges related to facilitating the cycle of scientific discovery through to the widespread adoption of a healthcare innovation have become of central concern to individuals and communities who seek or need healthcare; health professionals; policy makers; the funders of health services. Indeed, the interface between identifying knowledge needs for health improvement, pure scientific bench research, clinical trial based research, and, ultimately, the implementation of the results of research into some form of pragmatic outcome is a growing source of ongoing angst in both the research and clinical communities. It is a vital enterprise that, if achieved successfully, has the potential to result in dramatic improvements in global health outcomes. Whilst the translation of evidence into action is the raison d'être of the evidence-based practice movement, so, too, is it the core interest of translation science. Clarifying the nature and components of these two seemingly different (but, in our view, clearly complimentary) fields of endeavour and reconceptualizing this complementarity is important in advancing health policy and practice towards improving the health of people globally.

Nursing in central to the delivery of healthcare and an increasingly major contributor to the evidence-based practice movement broadly and the achievement of evidence-based practice in healthcare settings. Although the origins of evidence-based practice are in medicine, nursing is increasingly playing a role, particularly with respect to aligning practice with evidence at the point of care. Nurse scientists, therefore, are well positioned to take a leadership role in the field of translational science.

## 2. Clarifying and Reconceptualizing Evidence-Based Healthcare

There are a number of models that attempt to represent the components of evidence-based healthcare to facilitate understanding, analysis, improvement, and/or the replacement of the process as it is currently conceived, purported and practiced, for example, the Ace Star Model of Knowledge Transformation [1]; the five stage model of evidence-based healthcare [2]; the work of Titler and Everett [3]; the Stetler Model of Research Utilization [4–7]. Dobrow et al. [8] have developed a conceptual framework for evidence-based decision making, and Pearson et al. [9] report on the development of the JBI model of evidence-based healthcare (JBI Model).

### 2.1. The JBI Model

The JBI Model is developmental and, building on frameworks that have evolved, was constructed out of experience within the evidence-based practice field; the emerging international work of the Joanna Briggs Institute and the international Collaborating Centers of the Joanna Briggs Collaboration; involvement in disseminating, implementing and evaluating evidence-based guidelines in clinical settings; an examination of the scientific and professional literature.

Evidence-based practice can be conceptualized as clinical decision making that considers the best available evidence; the context in which the care is delivered; client preference; and the professional judgment of the health professional. The JBI model of evidence-based healthcare depicts the four major components of the evidence-based healthcare process as:

- healthcare evidence generation;
- evidence synthesis;
- evidence/knowledge transfer;
- evidence utilization.

**Figure 1:**
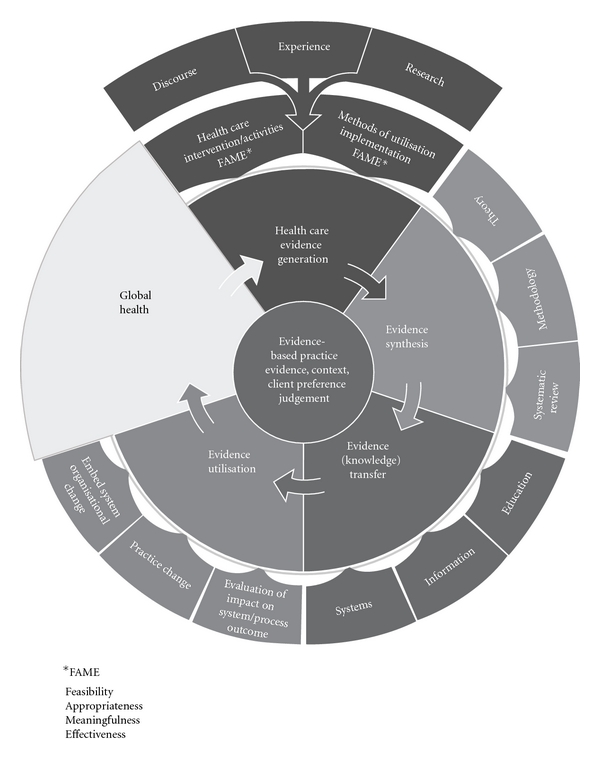
The JBI Model of Evidence-Based Healthcare [9].

Each of these components is modelled to incorporate their essential elements, and the achievement of improved global health is conceptualized as both the goal or endpoint of any or all of the model components and the raison d'être and driver of evidence-based healthcare (Figure 1).

Evidence-based healthcare is described as a cyclical process that derives questions, concerns, or interests from the identification of global healthcare needs by clinicians or patients/consumers and then proceeds to address these questions by generating knowledge and evidence to effectively and appropriately meet these needs in ways that are effective, feasible, and meaningful to specific populations, cultures, and settings. This evidence is then appraised, synthesized, and transferred to service delivery settings and health professionals who then utilize it and evaluate its impact on health outcomes, health systems, and professional practice.

The term “evidence” is used in the model to mean the substantiation or confirmation that is needed in order to believe that something is true [10]. Health professionals seek evidence to substantiate the worth of a very wide range of activities and interventions and thus the type of evidence needed depends on the nature of the activity and its purpose. The model depicts the process that the Joanna Briggs Institute uses to frame the provision of the best available evidence as well as utilization resources for health professionals to improve global health.

### 2.2. Evidence-Based Practice

Central to the JBI understanding of evidence-based practice is that health professionals will use research evidence together with the context of care, patient/client values and preferences, and the experience, expertise, and clinical judgment of the health professional. Using all of this information, health professionals are in a position to make evidence informed decisions.

### 2.3. Global Health

The model is premised on the belief that global health issues are both the driver and reason for evidence-based practice. The US Institute of Medicine (IOM) describes global health as “the goal of improving health for all people in all nations by promoting wellness and eliminating avoidable disease, disability, and death” [11]. For the purpose of this paper, global health issues are determined to be those as identified by health professionals working at the point of care or patients and consumers of health services. These issues are addressed through the generation of research evidence related to effectiveness, appropriateness, feasibility and meaningfulness for specific populations, cultures and settings.

The JBI model assumes that the raison d'être of the research enterprise is to address unmet needs for knowledge; that is, to identify and address concerns that arise out of the experiences of patients/clients, the users of healthcare, healthcare professionals, and families, carers, and communities to generate evidence that will effectively and appropriately meet these identified needs [9].

### 2.4. Healthcare Evidence Generation

 The model asserts that evidence may derive from experience, expertise, inference, deduction, or the results of rigorous inquiry but recognizes that “the results of well-designed research studies grounded in any methodological position are seen to be more credible as evidence than anecdotes or personal opinion” [9]. However, when no research evidence of this level exists, other evidence may represent the “best available evidence” for a specific question. This position is taken to provide the most meaningful and useful information to inform healthcare delivery. The JBI model also recognizes that health professionals consider evidence broader than evidence of effectiveness to inform their everyday practice [9] and that they are interested in evidence of feasibility, appropriateness, meaningfulness and/or effectiveness (FAME). Feasibility is the extent to which an activity is practical and practicable; appropriateness relates to the extent to which an intervention or activity fits with or is apt in a situation; meaningfulness refers to how an intervention or activity is experienced by the patient; effectiveness is the extent to which an intervention, when used appropriately, achieves the intended effect [9].

### 2.5. Evidence Synthesis

Evidence synthesis is the evaluation or analysis of research evidence and opinion on a specific topic to aid in decision making in healthcare. Although the science of evidence synthesis has developed most rapidly in relation to the meta-analysis of numerical data linked to theories of cause and effect, the further development of theoretical understandings and propositions of the nature of evidence, and its role in healthcare delivery and the facilitation of improved global health is identified as an important element of this component of the model. Similarly, the increasing, ongoing interest and theoretical work on methods of synthesizing evidence from diverse sources are depicted as an element of evidence synthesis.

The third element of evidence synthesis is the operationalization of methods of synthesis through the systematic review process. This element in the model is grounded in the view that evidence of feasibility, appropriateness, meaningfulness, effectiveness, and economics are legitimate foci for the systematic review process; and that diverse forms of evidence (from experience, opinion, and research that involves numerical and/or textual data) can be appraised, extracted, and synthesized [12].

There are three elements of synthesis in the model: theories that underpin synthesis, synthesis methodologies and the systematic review of evidence.

### 2.6. Evidence Transfer

This component of the model relates to the act of transferring evidence (knowledge) to individual health professionals, health facilities, and health systems globally by means of journals, other publications, guidelines, electronic media, education and training, and decision support systems. Evidence transfer is seen to involve more than disseminating or distributing information and should include careful development of strategies that identify target audiences—such as clinicians, managers, policymakers and consumers—and methods to package and transfer information that is understood and used in decision making. The model therefore depicts three major elements of evidence/knowledge transfer—education and training, information delivery, and the transfer of evidence though organizational and team systems. [9].

### 2.7. Evidence Utilization

This component of the model relates to the implementation of evidence into practice, as is evidenced by practice and/or system change. It identifies three elements: evaluating the impact of the utilization of evidence on the health system, the process of care and health outcomes; practice change; embedding evidence through system/organizational change.

The JBI Model of evidence-based healthcare adopts a pluralistic approach to the notion of evidence whereby the findings of qualitative research studies are regarded as rigorously generated evidence and other text derived from opinion, experience, and expertise is acknowledged as forms of evidence when the results of research are unavailable. Pearson and Jordan [13] say “While considerable work is being undertaken internationally with regard to translational research, an inclusive approach that accounts for all elements of the research cycle is yet to be developed and implemented in a systematic way in many countries”. They go on to link addressing these three gaps with the JBI model of evidence-based healthcare (JBI Model) described by Pearson et al. [9].

## 3. Clarifying and Reconceptualizing Translation Science

The Agency for Healthcare Research and Quality (AHRQ) in the United States report to congress stated that, “the ultimate goal (of AHRQ) is research translation—that is, making sure that findings from AHRQ research are widely disseminated and ready to be used in everyday healthcare decision making.” In 1999, AHRQ published its first Translating Research into Practice (TRIP) initiative. The purpose of the TRIP initiative was to generate new knowledge about approaches that promote the utilization of rigorously derived evidence to improve patient care. The Agency's goal was to enhance the use of research findings, tools, and scientific information that would work in diverse practice settings, among diverse populations, and under diverse payment systems [14]. The need to improve the translation of basic and fundamental research findings into routine clinical practice was also one of the main observations of the “Review of UK Health Research Funding” [15].

**Figure 2:**
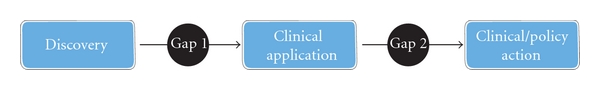
Two translation gaps in healthcare knowledge.

Knowledge translation has been seen as the process from basic discovery (basic/laboratory science) to intervention development (clinical trials) [16, 17], known as gap 1, translation 1, or T1; development (proven interventions) to delivery (used in practice) [17, 18], known as gap 2, translation 2, T2, or the know-do gap (Figure 2) [16, 18]. These gaps are two major obstacles in knowledge translation [17].

Mode 1 and Mode 2 knowledge have been used to describe different ways of knowledge generation. Whereas “Mode 1 relates to the traditional paradigm of scientific discovery” [19, page 225]. Mode 2 involves active involvement and collaboration of all stakeholders in terms of methodological development related to how to communicate knowledge and how to articulate the research questions. Mode 2 knowledge is seen as reflexive and transdisciplinary [19].

**Figure 3:**
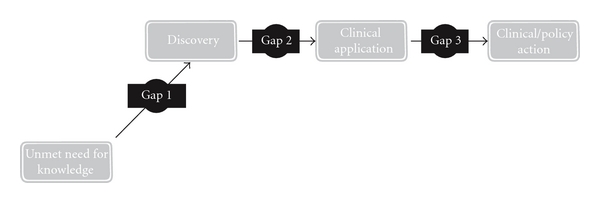
Three translation gaps in healthcare knowledge ([13], 2010).

The notion of translation gaps in the research-into-action cycle is common in all of the work in progress internationally, and Pearson and Jordan [13] suggest that there are essentially three critical gaps associated with the translation of research into action to improve outcomes and services (Figure 3).

### 3.1. Gap 1—From Knowledge Need to Discovery

The first gap relates to the gap between “knowledge needs” (as identified by patients, the community, clinicians, governments, and organizations) and the work undertaken by scientists and researchers during the “discovery” process. Within this gap there can be an integrated approach to topic selection, where there is active collaboration between those conducting the research and the end users of research (clinicians, patients, community). This gap is a vital component of translational research and is well addressed by very few groups, a notable exception being the National Institute for Health Research in the UK, with its associated Clinical Research Networks and its community engagement program “INVOLVE.”

### 3.2. Gap 2—From Discovery to Clinical Application

The second commonly identified gap relates to the gap between what is referred to here as “discovery research” (theoretical, epidemiological, or “bench” style research) and “clinical research” (experimental trials including but not limited to drug trials). This gap is the most commonly addressed gap on the international stage with significant work being undertaken in many countries; but for most, this is where translational research begins and ends.

### 3.3. Gap 3—From Clinical Application to Action

The third translation gap, that of translating research into practice, is rarely represented by strong programs in most countries, although some have recently ventured into this realm, notably in cardiology and metabolic/human nutrition centers.

Translating knowledge into action within healthcare is a complex, evolving, and dynamic process. While various models have been described, an accepted standard approach has yet to be widely adopted. Regardless of the model used, it is clear that three main gaps exist:

1. the gap between the need for knowledge and the discovery of that new knowledge;
2. the gap between the discovery of new knowledge and the clinical application of that knowledge;
3. the gap between the clinical application and the development of routine clinical actions or policy.

Pearson and Jordan [13], Pearson et al. [9, 20] and Pearson etal. [21], in drawing on the emerging literature, examine the relationship between the translation science cycle and evidence-based healthcare and suggest that the two processes are closely related and are complementary to each other.

## 4. Clarifying and Reconceptualizing the Relationship between Evidence Based-Healthcare and Translation Science

**Figure 4:**
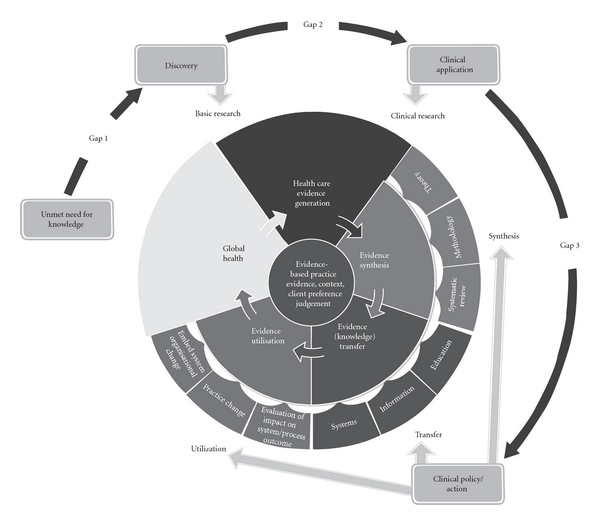
The relationship between the translation science cycle and evidence-based healthcare.

Pearson et al. [21] assert that the three translation gaps and the elements of the JBI model complement each other in modelling the relationship between the translation science cycle and the pragmatic evidence-based healthcare cycle (Figure 4).

The gap between the need for knowledge and discovery (gap 1) equates with the elements in the JBI Model that focus on the state of global health and the generation of knowledge through the conduct of basic or discovery-oriented research. Applying the findings of discovery research to the “real-world” (gap 2) through the conduct of clinical research (both trials and other health-related research including program evaluation and qualitative inquiry) is also a component of evidence generation. Evidence synthesis, transfer, and utilization in the JBI model represent the processes that most adequately address the gap between clinical application and clinical or policy action (gap 3).

## 5. Conclusion

Although evidence-based healthcare is gaining acceptance globally, it is complex, sometimes misunderstood, and frequently maligned. The sources of evidence accessed by practitioners, regardless of its nature—numerical, qualitative, or anecdotal—or its focus—feasibility, appropriateness, meaningfulness, or effectiveness—influences healthcare practice in all disciplines. Research evidence that is rigorously generated, regardless of design, demands due consideration of its quality prior to its utilization in the clinical environment. Evidence that is generated through the conduct of clinical trials; epidemiology; observational studies; qualitative studies; and action-oriented research are essential in addressing the knowledge and evidence needs of individuals and communities and of clinical and policy decision makers [20].

The JBI model of evidence-based healthcare emphasizes the need for the generation, synthesis, transfer, and utilization of evidence derived from diverse research approaches; has been constructed to facilitate reasoning and critique about evidence-based healthcare and its role in improving global health, within a logical conceptual framework.

Translation science (or translational research) is as complex and as frequently misunderstood as evidence- based healthcare. The dominant view of translation science overly emphasises the translation of the results of “basic,” “bench,” or discovery research into clinical application through the conduct of clinical trials—an enterprise that is now well entrenched in most advanced economies. We contend that translation is much more than the conduct of clinical trials to test discoveries. It begins with translating the questions that arise out of the need for knowledge in the “real world” into discovery research (addressing what we describe as gap 1); translating the findings of discovery research into clinical or policy application through clinical or policy research (addressing what we describe as gap 2); translating the findings of clinical or policy research into action at the clinical or policy level (addressing what we describe as gap 3). Integrating these three translation gaps into a model of evidence based health appears, to us, to clarify and reconceptualize the complexities of improving health outcomes through translating knowledge into action.
